# Commentary: An experimental study of gender and cultural differences in hue preference

**DOI:** 10.3389/fpsyg.2015.01840

**Published:** 2015-12-01

**Authors:** Christoph Witzel

**Affiliations:** Laboratoire Psychologie de la Perception, Université Paris DescartesParis, France

**Keywords:** color preferences, gender differences, cultural differences, biological mechanisms, color perception

Color preferences are a curious phenomenon. At first view, they seem to depend on the idiosyncrasies of individual observers and culture. At the same time, color preferences also involve regularities that are stable across individuals and cultures. In this way, color preferences highlight how seemingly arbitrary cognitive judgments are shaped by determinants that are not arbitrary at all.

In a recent study, Al-Rasheed (2015) has demonstrated the dependence of color preferences on culture and gender by comparing English and Arabic observers. The article concludes that “color preference varies with both sex and culture and that sex differences in color preference also vary culturally” (p. 4). While the observation of cultural and sex-specific differences is very much in line with previous studies, the conclusion that sex differences vary by culture is not.

Although it is true that some previous studies failed to identify systematic effects of gender on color preferences within cultures (for a brief review see Al-Rasheed, 2015, pp. 1–2; see also Palmer and Schloss, 2010); those studies did not test for cross-cultural patterns of sexual differences. In contrast, a recent study compared English and Chinese color preferences and found strong similarities in sex differences across these two fundamentally different cultures (Hurlbert and Ling, 2007). At the same time, another cross-cultural study (Taylor et al., 2013) contradicted those findings and suggested that sex differences varied between British observers and the Himba, a non-industrialized culture in rural Namibia. However, some of the cross-cultural effects observed in that study (Taylor et al., 2013) might be due to effects of measuring color preferences of non-industrialized remote observers on a computer screen (Sorokowski et al., 2014).

A recent study introduced a new approach to test for similarities in sex differences across cultures (Sorokowski et al., 2014). This study compared color preferences in Polish participants to those of the Yali, a remote, non-industrialized tribal community in the mountain area of Papua, Indonesia (Sorokowski et al., 2014). In that study, Papuan observers had also very different color preferences than Polish observers, and there were pronounced differences across the sexes. Most importantly, however, this study revealed a very strong cross-cultural similarity in the way in which the color preferences of women and men differed. In particular, sexual contrasts were calculated as the differences between the preferences of women and men, for Polish and Papuan observers separately. The Polish and Papuan sexual difference were highly correlated (*r* = 0.93, *p* < 0.01). This finding showed that “sex differences in color preferences transcend extreme differences in culture and ecology” (Sorokowski et al., 2014).

One limitation of the study of Sorokowski et al. (2014) was that they had a limited stimulus set of 12 colors, and the question arose whether their results depended on their particular set of stimuli. The data of Al-Rasheed (2015) for color preferences in English and Arabic women and men may be used to test this finding with a different set of eight colors. Moreover, since Al-Rasheed (2015) used the same color stimuli as Hurlbert and Ling (2007), their data can be directly compared. For this reason, I reanalyzed the data from Al-Rasheed's (2015, Figures 1B,C) and Hurlbert and Ling's (2007, Figure 1) study by calculating the sexual contrasts (preferences of women—preferences of men) per color for Arabic, English, and Chinese observers measured in these two studies. Figure 1A shows the results.

**Figure 1:**
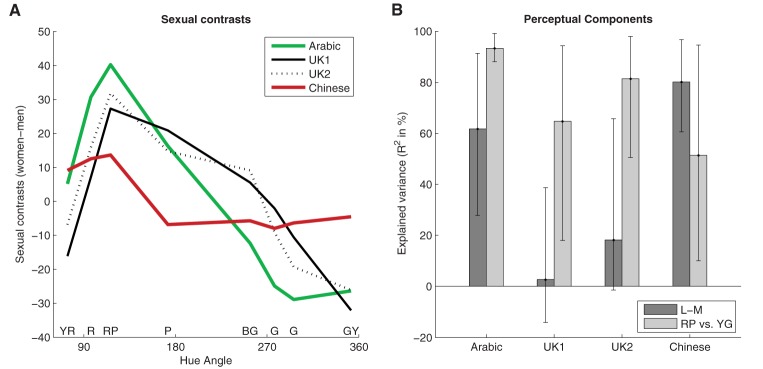
**Sexual contrasts in Al-Rasheed's (2015) and Hurlbert and Ling's (2007) study**. **(A)** Sexual contrasts (average preferences of women—average preferences of men) along the y-axis as a function of CIELUV hue angle in degree for Arabic (green curve; 36 women and 32 men), and English observers (solid black curve; 31 women and 17 men) in Al-Rasheed's (2015) study, and for English (dotted black curve; 92 women and 79 men) and Chinese (red curve; 18 women and 19 men) observers in Hurlbert and Ling's (2007) study. **(B)** Variance of sexual contrasts across colors explained by the biological component model (L-M). Error bars show 90% confidence interval (i.e., 95% for one-tailed tests, as here) based on boot-strapping of the correlation coefficient r (Pernet et al., 2012).

First, I tested whether the data in Figure 1A reproduces the positive correlation between sexual contrasts across cultures found previously by Sorokowski et al. (2014). The pattern of sexual contrasts across stimuli was very similar between Arabic (green curve) and English observers from both studies (UK1 and UK2: solid and dotted black curves). Correlations across the *n* = 8 colors were calculated between the sexual contrasts of the different cultural groups (i.e., the different curves in Figure 1A), and right-tailed t-statistics are reported to test whether there is a positive correlation. As would be expected, English observers in both studies had almost the same sexual contrast (*r* = 0.93, *p* < 0.001), which illustrates that the two studies can be directly compared to each other. Despite the low number of cases (*n* = 8), the sexual contrasts of Arabic observers were significantly positively correlated with those of the English observers from Al-Rasheed's (2015) study (*r* = 0.73, *p* = 0.02), the English observers from Hurlbert and Ling's (2007) study (*r* = 0.89, *p* = 0.002), and those of the Chinese observers (*r* = 0.80, *p* = 0.01). The correlation between the sexual contrast of the Chinese and English observers of Hurlbert and Ling's (2007) study were only marginally significantly correlated (*r* = 0.56, *p* = 0.08). Finally, the correlation between Chinese and the English observers from Al-Rasheed's (2015) study was also positive, but did not reach significance (*r* = 0.29, *p* = 0.25). These results were confirmed through robust correlation analyses (Pernet et al., 2012); for further details see Figures S1, S2 of the Supplementary Material. Taken together, these results further support the finding of trans-cultural patterns of sexual dimorphism reported by Sorokowski et al. (2014).

Hurlbert and Ling (2007) demonstrated for their data that preference differences between men and women, across two cultures, are largely captured by differences in weighting on a low-level sensory dimension of color vision, the L-M mechanism, which they called “biological components of sex differences in color preference.” In contrast, Sorokowski et al. (2014) argued, that sexual contrasts must necessarily correlate with any perceptual dimension of color vision, not only the biological components, because sexual contrasts change gradually across colors. To illustrate this idea, they showed that sexual contrasts may be still better modeled by a simple dimension of hue similarity than by the L-M mechanism. For this purpose, they modeled hue differences as the relative difference in hue of each stimulus color from the color with the minimum and maximum sexual contrast. I did the same test for the data in Figure 1A. I determined the colors that correspond to the minimum and maximum of the sexual contrasts averaged across the samples of the four groups of participants. The color with maximum sexual contrast (i.e., more preferred by women than by men) was the color RP, and the minimum (i.e., more preferred by men) the color GY. Note that the hue difference between these colors does not coincide with the dimension of the L-M mechanism. As in Sorokowski et al. (2014), I determined the relative hue difference as the angular difference divided by the difference between the colors with minimum and maximum sexual contrasts. Figure 1B provides the variance explained by the correlations between sexual contrasts (i.e., the curves in Figure 1A) and the weights of the stimuli along the L-M mechanisms on the one hand (dark bars), and the variance explained by the correlation between the sexual contrasts and the relative hue difference on the other (light bars). The hue differences were significantly correlated with the sexual contrasts of all groups. At the same time, the L-M component did not correlate with the English sexual contrasts of both studies (UK1 and UK2). For details see Table 1 of the Supplementary Material. The hue differences explained marginally significantly more variance of sexual contrasts than the “biological component” for Arabic and the two English groups of observers (all *z* < −1.5; *p* < 0.07; with *t*-tests based on Fisher transforms). Only the sexual contrasts of Chinese observers were (non-significantly) better modeled through the biological component than by the hue difference. Consequently, only the sexual differences of Chinese were in line with Hurlbert and Ling's (2007) claim that sexual dimorphism in color preferences are specifically related to “biological components.”

In sum, the re-analyses of Al-Rasheed's (2015) data revealed positive correlations of sexual contrasts across cultures, and hence extends previous findings to different cultures and different samples of colors. These findings strongly support the idea that “sex differences in color preferences transcend extreme differences in culture and ecology” (Sorokowski et al., 2014). Due to the small sample of colors, statistics did not allow to draw a firm conclusion about whether these cross-cultural regularities are specific to the “biological components” of color vision (Hurlbert and Ling, 2007), or not (Sorokowski et al., 2014). The present findings are further supported by a recent study with more stimuli (32) that showed a cross-cultural correlation between sexual contrasts of American and Japanese, and that found little evidence in support for the “biological components” (Yokosawa et al., 2015). Systematic cross-cultural comparisons with larger samples of colors are necessary to find out whether sex differences are universal or common to some but not all cultures, as Taylor et al.'s (2013) data arguably suggests.

Overall, the present findings suggest that there are determinants of color preferences that transcend cultural boundaries, and they encourage further research to identify what the exact cross-cultural determinants of sexual dimorphism are. Because red-green dichromacy modulates color preferences (Álvaro et al., 2015), the observed transcultural sexual differences in color preferences might be linked to the sexual differences in the prevalence of red-green dichromacy.

## Conflict of interest statement

The author declares that the research was conducted in the absence of any commercial or financial relationships that could be construed as a potential conflict of interest.
