# Supplementary material for: Commentary: An experimental study of gender and cultural differences in hue preference
Source: Front Psychol. 2015 Dec 1;6:1840. doi: 10.3389/fpsyg.2015.01840 (PMC4664724; doi:10.3389/fpsyg.2015.01840)
Supplement: Supplementary file 1 [file Presentation1.PDF]

Witzel, C. (2015). **Commentary: "An experimental study of gender and cultural differences in hue preference"**.
Frontiers in Psychology, *6*. doi: 10.3389/fpsyg.2015.01840, http://journal.frontiersin.org/article/10.3389/fpsyg.2015.01840/full

Supplementary Material

Content:

1. Correlations between sexual contrasts for different cultures

Figure S1: Robust correlation analyses for sexual contrasts across different cultures

Figure S2: Correlations between sexual contrasts with confidence intervals.

2. Correlations between perceptual coponents

Table S1: Correlations with perceptual components.

1. Correlations between sexual contrasts for different cultures

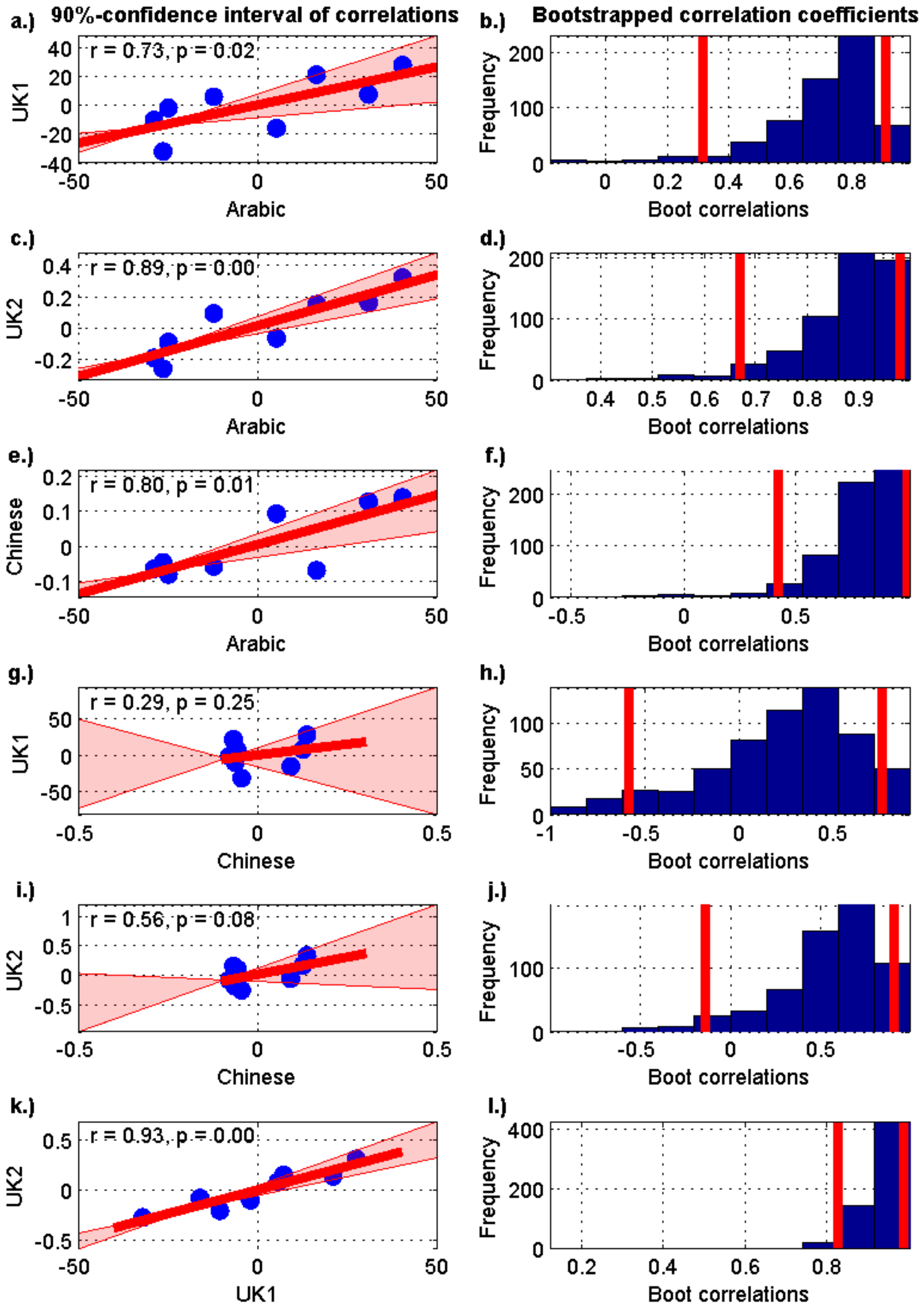

Figure S1. Robust correlation analyses for sexual contrasts across different cultures. The figure shows the estimation of bootstrapped confidence intervals for the Pearson correlation coefficient based on the robust correlation analyses toolbox of Pernet and colleagues (Pernet et al., 2012). The left side (panels a,c,e,g,I,k) shows scatterplots of the sexual contrasts for Arabic and English (a and c), Arabic and Chinese (e), Chinese and English (g and i), and the two samples of English observers along the x- and y-axis respectively. Blue disks depict the data for the 8 stimuli, the thick red line the regression line, and the desaturated area the bootstrapped confidence interval. The right side (panels b,d,f,h,j,l) illustrates the results of the bootstrapping. Bars show the frequency of correlation coefficients along the y-axis, vertical red lines the confidence intervals. Because the present analyses tested for a positive correlation 90%-confidence intervals were bootstrapped, which correspond to one-tailed 95% confidence intervals.

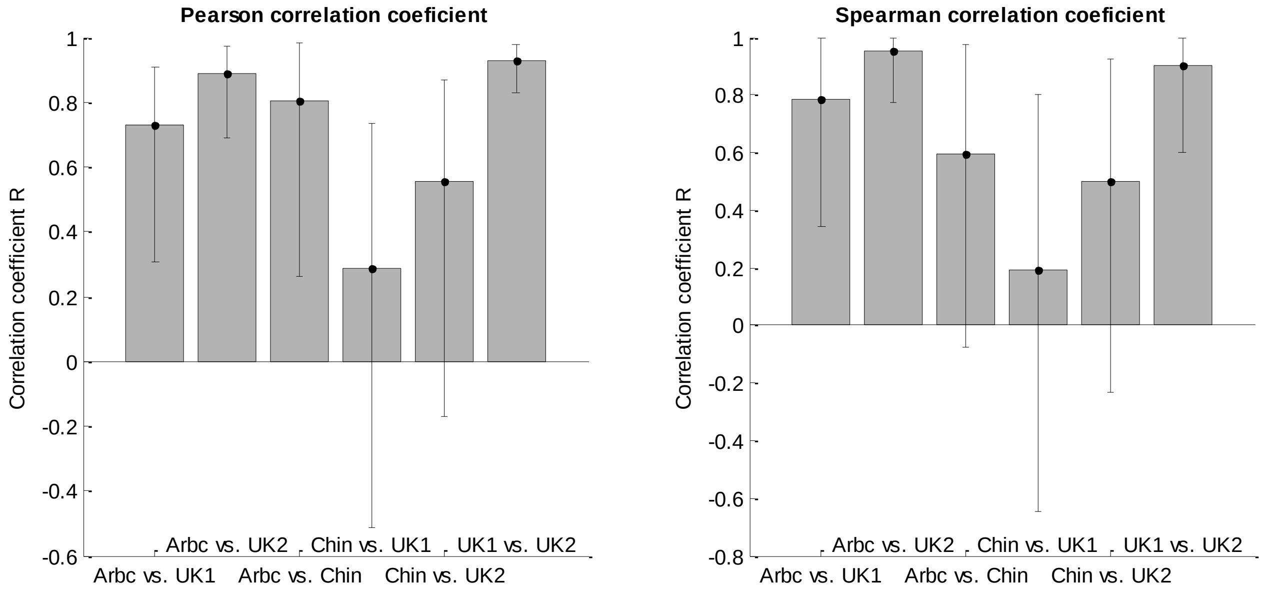

Figure S2. Correlations between sexual contrasts with confidence intervals. Panel a shows Pearson, panel b Spearman correlation coefficients. Each bar shows the correlation coefficients for all correlations between sexual contrass. The first bar corresponds to the correlation and confidence intervals illustrated in Figure S1. Arbc = Arabic, Chin = Chinese, UK1 = English from Al-Rasheed's (2015) study, and UK2 = English from Hurlbert and Ling's study (2007). Note that Pearson correlations that were significant according to the t-test as reported in the main article, were also significant according to confidence intervals based on boot-strapping. Moreover, Spearman (instead of Pearson) correlations also supported a positive relationship between sexual contrasts of Arabic observers and the two samples of British observers (r = 0.79, p = 0.01; and r = 0.95, p = 0.0001); the Spearman correlation between sexual contrasts of Arabic and Chinese observers was marginally significant (r = 0.60, p = 0.06).

2. Correlations with perceptual components

	Pearson correlations						Spearman correlations					
	r	t	p	CI		Sig	r	t	p	CI		Sig
**Arabic**												
L-M	0.79	3.1	0.01	0.54	0.95	1	0.57	1.7	0.07	0.04	0.90	0
RP-YG	0.97	9.2	< 0.0001	0.94	0.997	1	0.98	11.0	< 0.0001	0.80	1	1
**UK1**												
L-M	0.16	0.5	0.35	-0.40	0.66	0	0.07	0.2	0.43	-0.64	0.62	0
RP-YG	0.80	3.3	0.008	0.40	0.97	1	0.83	3.7	0.01	0.34	1	1
**UK2**												
L-M	0.43	1.2	0.29	-0.16	0.79	0	0.38	1.0	0.18	-0.31	0.80	0
RP-YG	0.90	5.1	0.001	0.70	0.99	1	0.98	11.0	< 0.0001	0.80	1	1
**Chinese**												
L-M	0.90	4.9	0.002	0.74	0.99	1	0.76	2.9	0.01	0.24	1	1
RP-YG	0.72	2.5	0.03	0.30	0.97	1	0.55	1.6	0.08	-0.20	1	0

Table 1. Correlations with perceptual components. The columns provide r = correlation coefficient, t = Students t-statistic, p the probability resulting from the right-tailed t-test, CI = confidence interval calculated through bootstrapping, and Sig the significance of the correlations according to bootstrapping following (Pernet et al., 2012).
